# Tie1 ectodomain cleavage governs vascular responses to inflammatory stimulation but is dispensable for angiogenic signaling

**DOI:** 10.1172/JCI204085

**Published:** 2026-10-01

**Authors:** Miki Kamiyama, Jingjing Shi, Lorna Rinck, Yanzhu Lin, Yifang Mao, Xiaowen Zhang, Ashik Ahmed Abdul Pari, Stephanie F. Preuss, Marlene Hoffarth, Gladys Hofsetz, Yuxi Di, Asmaa Zahran, Carleen Spegg, Maria Riedel, Donato Inverso, Roxana Ola, Junhao Hu, Kari Alitalo, Hellmut G. Augustin, Mahak Singhal

**Affiliations:** 1 European Center for Angioscience, Medical Faculty Mannheim, Heidelberg University, Mannheim, Germany.; 2 German Cancer Research Center, Heidelberg, Germany.; 3 Helmholtz Institute for Translational AngioCardioScience, Max Delbrück Center, Heidelberg University, Heidelberg, Germany.; 4 Shanghai Institute of Organic Chemistry, Chinese Academy of Sciences, Shanghai, China.; 5 Wihuri Research Institute, University of Helsinki, Helsinki, Finland.

**Keywords:** Inflammation, Vascular biology, Angiogenesis, Signal transduction

## Abstract

Ectodomain cleavage of the vascular receptor Tie1 distinguishes the inflammatory from the angiogenic functions of Angiopoietin-Tie signaling, mechanistically uncoupling two fundamental vascular programs.

**To the Editor:** Interactions between leukocytes and endothelial cells (ECs) govern the host response to pathological challenges. Yet, early molecular events sensitizing ECs to inflammatory stimuli remain elusive. Among EC-specific pathways, Angiopoietin (Ang)/Tie signaling not only regulates vascular development and maturation but also orchestrates endothelial activation during inflammation (1). The Tie1 receptor, lacking high-affinity binding to Ang1 or Ang2, is proteolytically cleaved, with its extracellular domain (ECD) shed into the circulation during inflammation (2, 3). Employing genetically engineered cleavage-resistant (Tie1-CR) mice, we investigated the functional impact of Tie1-ECD cleavage, distinguishing angiogenic and inflammatory responses.

Homology analysis and site-specific mutagenesis identified R747 as an inflammation-induced cleavage site in mouse Tie1 (Supplemental Figure 1, A–D; supplemental material available online with this article; https://doi.org/10.1172/JCI204085DS1). We genetically modified nucleotides, encoding R747, to establish a targeted mutant mouse line. Western blotting with lung tissues of Tie1-CR mice showed no Tie1-ECD band, confirming the CR status of Tie1 (Supplemental Figure 1E). Tie1-CR mice were born without developmental abnormalities in the expected Mendelian ratio (Figure 1A).

Postnatally, Tie1 is essential for vascular development, as inhibition of Tie1 blunted angiogenesis in retinas or tumors (4–6). Surprisingly, the retinal vasculature developed normally in Tie1-CR mice, with unaltered vascular outgrowth, tip cells, and pericyte coverage (Figure 1, B and C, and Supplemental Figure 1, F and G). Likewise, lung EC proliferation and dermal lymphatics were comparable between genotypes (Supplemental Figure 1, H and I). Longitudinal monitoring revealed comparable growth and no difference in lifespan (Figure 1D, Supplemental Figure 1, J and K, and Supplemental Table 1), suggesting no physiological defects during development, adulthood, and aging in Tie1-CR mice.

Interrogating the effects of Tie1 cleavage on Ang1-Tie2 signaling, we challenged mice with AAV-expressing COMP-Ang1. Ang1-stimulated phosphorylation of Tie2 and downstream Akt, and Tie1–Tie2 interaction were not different between Tie1-CR and Tie1-WT mice (Supplemental Figure 1, L and M). Similarly, Tie1-CR and Tie1-WT mice showed comparable tumor growth, intratumoral blood and lymphatic vessel area, and pericyte-covered blood vessels (Figure 1E and Supplemental Figure 2, A–C), corroborating that Tie1 cleavage is dispensable for angiogenesis-regulating functions.

Next, we compared transcriptomic profiles of lung ECs from adult Tie1-CR and Tie1-WT mice (Supplemental Figure 2, D and E). Differentially expressed genes (755 up- and 509 downregulated in Tie1-CR ECs) corresponded to inflammation-related bioprocesses and a reduced vascular responsiveness to stimuli, such as LPS and TNF-α (Figure 1, F and G, and Supplemental Figure 2, F and G). We therefore hypothesized that resisting Tie1 cleavage may suppress inflammation-triggered vascular activation.

Experimentally, TNF-α stimulation led to a cleavage of Tie1 and accumulation of Ly6G^+^ myeloid cells in the lungs of Tie1-WT mice (Figure 1, H–K, and Supplemental Figure 2, H–J). In contrast, Tie1-CR mice resisted TNF-α–induced Tie1 cleavage and displayed reduced lung infiltration of Ly6G^+^ myeloid cells. Molecular analyses revealed higher phosphorylation of Tie2 and Akt, as well as Tie1–Tie2 interaction, along with lower nuclear localization of FOXO1 in lungs of Tie1-CR mice after TNF-α stimulation (Supplemental Figure 3, A–C). Similarly, LPS stimulation led to Tie1 cleavage and lung infiltration of Ly6G^+^ myeloid cells in Tie1-WT mice (Supplemental Figure 3, D–G). However, LPS-induced Tie1 cleavage and lung myeloid cell emigration were blunted in Tie1-CR mice. Consequently, male and female Tie1-CR mice survived longer compared with Tie1-WT mice after LPS administration (Figure 1L). Mechanistically, while LPS induced comparable early endotoxemic responses (Supplemental Figure 3H and Supplemental Figure 4, A and B), Tie1-CR lung tissues had higher phosphorylated Tie2 and Akt, along with lower nuclear FOXO1, 16 hours after LPS administration (Supplemental Figure 4, C–E). Together, these data suggest that Tie1 cleavage regulates inflammation-induced loss of Tie2 phosphorylation and vascular stability.

Lastly, cellular assays revealed a reduced capacity of TNF-α–induced transendothelial migration of leukocytes across Tie1-CR ECs, whereas leukocyte adhesion to ECs remained comparable (Figure 1M). Similarly, tumor cells showed reduced transendothelial migration across Tie1-CR ECs (Supplemental Figure 4F). Concomitantly, Tie1-CR mice harbored fewer lung metastatic tumors following intravenous injection of melanoma cells (Figure 1N and Supplemental Figure 4G).

In conclusion, the present study demonstrates a crucial role of Tie1 cleavage in sensitizing the vascular endothelium to inflammatory challenges and controlling the entry of leukocytes into the lungs. By specifically targeting ectodomain cleavage, we dissect the inflammatory functions of Tie1 from its roles in facilitating angiogenesis. Overall, the data shed fundamental insight into the gatekeeping mechanisms of the vasculature and reveal Tie1 cleavage as a potential opportunity to on-demand stimulate a vascular immunomodulatory response.

For detailed methods, information regarding sex as a biological variable, statistics, study approval, data availability, and author contributions, see the supplemental materials.

## Conflict of interest

The authors have declared that no conflict of interest exists.

## Funding support

- DFG grant CRC1366 “Vascular control of organ function” (project 394046768); projects B07 to RO, C05 to HGA, and C06 to MS.
- DFG grant “Endothelial NFKBIZ in tumor progression and metastasis” (project 510602219) to MS.
- European Research Council Advanced Grant “AngioMature” (project 787181) to HGA.
- European Research Council Starting Grant “Vascular-Rhythm” (project 101220624) to MS.
- State of Baden-Württemberg Grant “Innovation Campus Health + Life Science Alliance Heidelberg Mannheim” to MS.
- China Scholarship Council to YM.
- Naito Foundation, Nakatomi Foundation, and DKFZ Heidelberg fellowships to MK.

## Supplementary Material

Supplemental data

Unedited blot and gel images

Supporting data values

## Figures and Tables

**Figure 1:**
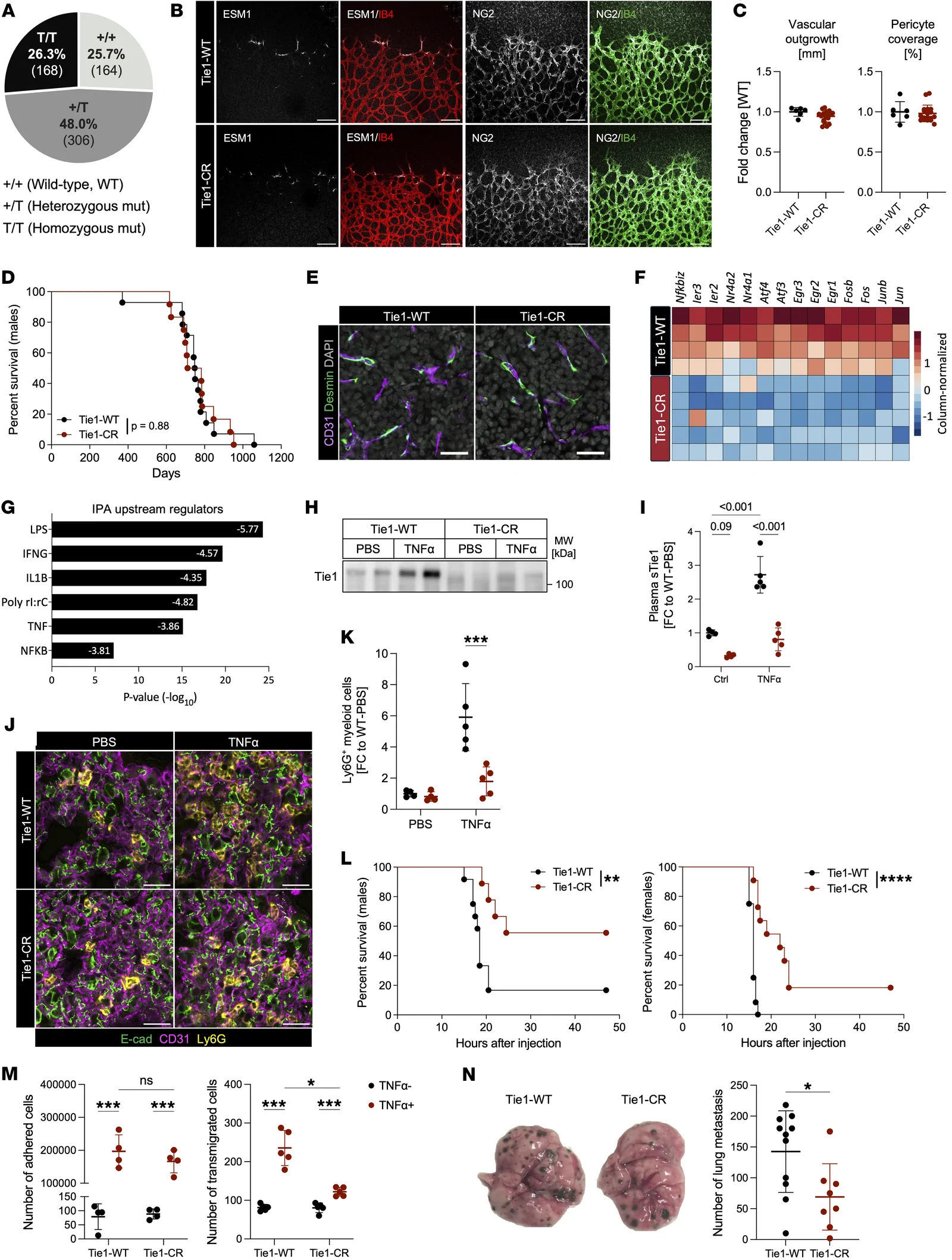
Tie1 cleavage is dispensable for angiogenic responses but critical for inflammatory vascular responses. (**A**) Genotype distribution of offspring from heterozygous breeders. T, targeted mutation. (**B** and **C**) Retinal analysis (P6): representative images (**B**) stained with EC marker IB4 (isolectin B4), tip cell marker ESM1 (endothelial cell–specific molecule 1), and/or pericyte marker NG2 (chondroitin sulfate proteoglycan 4) and quantification (**C**) of vascular outgrowth and pericyte coverage. Tie1-WT, *n* = 6; Tie1-CR, *n* = 18 mice. Scale bars: 100 μm. (**D**) Kaplan-Meier plot displaying lifespan of males. Tie1-WT, *n* = 14; Tie1-CR, *n* = 12 mice. (**E**) Representative images of Lewis Lung Carcinoma tumor vessels stained with EC marker CD31 and pericyte marker desmin. Scale bars: 50 μm. (**F**) Heatmap showing immune-responsive genes corresponding to the enriched gene set “TNF-alpha signaling via NF-κB.” (**G**) Top-ranked upstream regulators negatively predicted with the Ingenuity Pathway Analysis platform in Tie1-CR ECs. (**H** and **I**) IB (**H**) and quantification (**I**) of Tie1-ECD in plasma from Tie1-WT or Tie1-CR mice challenged with TNF-α or PBS for 6 hours. PBS, *n* = 4; TNF-α, *n* = 5 each. sTie1, soluble Tie1; FC, fold change. (**J** and **K**) Representative images (**J**) and FACS-based quantification (**K**) of Ly6G^+^ myeloid cells in lungs challenged with TNF-α or PBS for 6 hours. PBS, *n* = 3 (**J**) and 4 (**K**); TNF-α, *n* = 5 each (**J** and **K**). Scale bars: 20 μm. (**L**) Survival curves following LPS-induced endotoxemia. Males: Tie1-WT, *n* = 12; Tie1-CR, *n* = 9 mice. Females: Tie1-WT, *n* = 12; Tie1-CR, *n* = 11 mice. (**M**) Adhesion (left) and transendothelial migration (right) of U937 monocytes to Tie1-WT/Tie1-CR ECs. *n* = 4 (adhesion) and *n* = 5 (migration) replicates. (**N**) Representative images and the number of metastatic nodules in the lungs 2 weeks after i.v. injection of B16F10 cells. Tie1-WT, *n* = 11; Tie1-CR, *n* = 8 mice. Data are presented as mean ± SD (**C**, **I**, **K**, **M**, and **N**). ****P < 0.0001, ***P < 0.001, **P < 0.01, *P < 0.05. Mann-Whitney *U* test (**C** and **N**), 2-way ANOVA followed by Šidák’s multiple-comparison test (**I**, **K**, and **M**), and log-rank (Mantel-Cox) test (**D** and **L**).

## Version 1 10/01/2026

Electronic publication
