# Supplementary material for: Tie1 ectodomain cleavage governs vascular responses to inflammatory stimulation but is dispensable for angiogenic signaling
Source: J Clin Invest. 2026 Oct 1;136(19):e204085. doi: 10.1172/JCI204085 (PMC13626837; doi:10.1172/JCI204085)
Supplement: Supplementary file 1 [file jci-136-204085-s353.pdf]

Tie1 ectodomain cleavage governs the vascular response to inflammatory stimulation, but is dispensable for angiogenic signaling

Miki Kamiyama[1,2,3,4,#], Jingjing Shi[2,3,#], Lorna Rinck[1], Yanzhu Lin[5], Yifang Mao[1], Xiaowen Zhang[2,3,6], Ashik Ahmed Abdul Pari[2,3], Stephanie F. Preuss[2,3], Marlene Hoffarth[1,4], Gladys Hofsetz[1,4], Yuxi Di[5], Asmaa Zahran[1], Carleen Spegg[2,3], Maria Riedel[2,3], Donato Inverso[2,3,$], Roxana Ola[5], Junhao Hu[7], Kari Alitalo[8], Hellmut G. Augustin[2,3,*], Mahak Singhal[1,4,*]

Supplemental Data

Supplemental Methods and Figures

Supplemental methods

Animals

In this study, the following aged female and male mice were used - postnatal 6-7 days for developmental analysis, 3 weeks old for dermal lymphatic analysis, 5-16 weeks old for body weight measurement in basal state, from birth to reaching the humane endpoint criteria for aging cohort, and 8-25 weeks old for other experiments. All mice were housed on a 12-hour light/dark cycle with free access to food and drinking water in specific and opportunistic pathogen-free animal facilities. The mice from aging cohort were euthanized when humane endpoint criteria are met, according to animal caretakers.

Study approval

All animal experiments were approved by the governmental (G-199/15, G-257/18, G-9/19, G-124/21 and G-133/24 from Regierungspräsidium Karlsruhe, Germany) and institutional (DKFZ370, DKFZ434, I22-31, IRCBC-2018-001) Animal Care and Use Committees. All experiments were performed in accordance with the respective institutional guidelines for the care and use of laboratory animals.

Sex as a biological variable

This study examined both male and female animals, and the findings were similar for both sexes.

Generation of Tie1-CR mice

Previous *in vitro* experiments with human ECs predicted inflammation-driven proteolytic cleavage at the E749/S750 peptide bond in the juxta-membrane domain of human Tie1 (hTie1) (1). To map the cleavage site in mTie1, we initially pursued homology analysis comparing amino acid sequences of hTie1 and mTie1 (**Supplemental Figure 1A**). These *in silico* analyses led us to hypothesize that mTie1 would potentially be cleaved at the RESR motif (744-747 amino acids of mTie1). Next, we established site-specific mutants and identified the Tie1-R747 as a crucial peptide for Tie1-ECD cleavage. Building on these *in vitro* assays, we devised a CRISPR-based strategy to mutate the 747 amino acid (R → E), thereby generating a gene-edited mouse line. To this end, guide RNA (gRNA) sequences were designed with the CRISPR DESIGN tool (Zhang lab) to target the regions surrounding the Tie1 R747 codon (CGG) within the ECD of exon 14. Two single gRNAs with a higher score by CRISPR DESIGN were transfected together with a repairing DNA template. The Tie1-CR mouse line was generated in collaboration with the Transgenic Service at DKFZ, where the embryonic microinjection and embryo transplantation were performed. Cas9 mRNA together with the sgRNA and the repairing template were injected in the zygote. The descendants were backcrossed for at least three generations with C57BL/6N mice before experimental use. Genome DNA was extracted from ear punches for genotyping. Forward (5’-CTTCCACCTTACCCCCAACC-3’) and reverse (5’-GGACAGTTTCCCAGAGAGGC-3’) primers were used for the PCR reaction and the PCR samples were enzymatically digested with FastDigest MspI (Thermo Fisher Scientific, #FD0544) to recognize point mutation.

LLC primary tumor model

LLC cells (1×10^6 cells in PBS) were subcutaneously inoculated in Tie1-WT and Tie1-CR mice as previously described (2). Primary tumors were routinely measured in size with caliper. Tumors were collected at an average size of 300 mm^3. All the mice were routinely checked for the humane endpoint criteria; tumor with a diameter of >1.3 cm, 20% weight loss compared with body weight at the start

of the experiment, lack of food or water intake, obvious respiratory symptoms, abnormal crouched posture, apathy, immobility and anemia.

B16F10 experimental metastasis model

B16F10 cells ($2x10^5$ cells in PBS) were injected into the tail vein of Tie1-WT and Tie1-CR mice. Lungs were collected two weeks after tumor cell inoculation, and metastatic foci were imaged and counted under a stereomicroscope.

TNFα-induced inflammation

Mouse recombinant TNFα was injected into the tail vein (100 µg/kg body weight, BioLegend, #575208) of Tie1-WT and Tie1-CR mice. PBS was injected as a negative control. Mice were sacrificed at indicated time points. Blood was collected via heart puncture and lungs were collected and processed for flow cytometry analysis, embedded for stainings and/or snap frozen for protein isolation.

LPS-induced endotoxemia

Endotoxemia was induced by i.p. injection of LPS (18 mg/kg body weight for males, 15 mg/kg body weight for females, Sigma-Aldrich, #L4391) in Tie1-WT and Tie1-CR mice. PBS was injected as a negative control. When indicated, body temperature was monitored every hour with infrared thermometer. Mice were sacrificed at indicated time points, or when humane endpoint criteria were reached. Blood was collected via heart puncture and lungs were collected and processed for flow cytometry analysis, embedded for stainings and/or snap frozen for protein isolation.

COMP-Ang1 injection

Adeno-associated viral (AAV) vectors (control or overexpressing cartilage oligomeric matrix protein (COMP)-Ang1) were produced as previously described (3). Tie1-WT and Tie1-CR mice were injected with control AAV9-Empty vector or AAV9-COMP-Ang1 (10^{11} viral vector particles per mouse) intraperitoneally. Lungs were collected two weeks later and snap frozen for protein isolation.

Cell culture, transduction and treatment

B16F10, LLC, LLC-RFP and BEND3 cells (ATCC) were maintained with DMEM (high glucose, Thermo Fisher Scientific, #61965059), 10% FBS (Thermo Fisher Scientific, #10500064 or #A5256801), 1% non-essential amino acids (Thermo Fisher Scientific, #11140050) and 1% Penicillin-Streptomycin (Sigma-Aldrich, #P4333-100ML). U937-GFP cells were maintained in suspension with RPMI 1640 (Thermo Fisher Scientific, #A1049101), 10% FBS (Thermo Fisher Scientific, #10500064 or #A5256801) and 1% Penicillin-Streptomycin (Sigma-Aldrich, #P4333-100ML). All cells were cultured at 37°C with 5% CO_2 and routinely tested for mycoplasma by PCR. For overexpressing mTie1 constructs, BEND3 cells were seeded and transduced with the corresponding lentivirus (Table S2) plus 10 µg/ml polybrene (Millipore, #TR-1003-G), selected with 5 µg/ml puromycin (Thermo Fisher Scientific, #A11138-03) and maintained with 2 µg/ml puromycin. To induce Tie1 cleavage *in vitro*, BEND3 cells overexpressing mTie1 constructs were seeded, changed medium on the next day with 0.5% FBS or without FBS, and were exposed to stimulations such as PMA (10 ng/ml, Sigma-Aldrich, #P1585), mouse IL-1β (100 ng/ml, BioLegend, #575102) or mouse TNFα (100 ng/ml, BioLegend, #575202) for 1 hour. The cell supernatant was collected, and the cells were harvested for protein isolation or staining.

Postnatal retinal vasculature analysis

At P6, eyeballs were isolated from Tie1-WT and Tie1-CR pups and fixed in 4% paraformaldehyde (PFA) for 17 minutes at room temperature (r.t.). The eyes were dissected under a microscope and the retinas were isolated. The retinas were washed with PBS for 3 times and incubated with the primary

antibodies against ESM1 (R and D systems, #AF1999) and NG2 (Millipore, #AB5320) overnight at 4°C in blocking buffer (1% FBS, 3% BSA, 0.5% Triton X-100, 0.01% sodium deoxycholate in PBS at pH 7.2). Next day after washing, the retinas were incubated with the corresponding secondary antibodies (Invitrogen, #A-21206 and #A-21447) together with Isolectin GS-IB4 (Invitrogen, #I21412) in PBLEC buffer (1 mM $CaCl_2$, 1 mM $MgCl_2$, 1 mM $MnCl_2$ and 0.25% Triton X-100 in PBS at pH 7.2) for 1 hour at r.t., followed by post-fixation with 4% PFA for 40 minutes at r.t. The stained retinas were flattened by four radical incisions and their spherical structure was opened as a shape of four-leaf clover. The retinas were mounted in fluorescent mounting medium (CarlRoth, #HP19.1) and covered with a thin glass coverslip. The images of the retinas were taken using Zeiss LSM800 Confocal Laser Scanning Microscope, and image analysis was performed using ImageJ (RRID:SCR_003070). Vessel area was calculated as $IB4^+$ area per retina area. Vessel outgrowth reflects the average distance between the optic nerve and the outer rim of a vascularized retina leaf. Pericyte (PC) coverage was calculated as $NG2^+$ area per vessel area. The number of tip cells ($ESM1^+IB4^+$ cells) were counted per field. All values were normalized to the average of WT pups.

<u>Flow cytometry</u>

Isolation of lung endothelial cells

Lungs were dissociated into single cell suspension with Liberase digestion enzyme mix (Roche, #5401127001) plus DNAse I (Sigma-Aldrich, #10104159001) and subjected to erythrocyte lysis. ECs were enriched from lysed single cell suspensions using CD31 microbeads (Miltenyi Biotec, #130-097-418) and LS columns (Miltenyi Biotec, #130-042-401) following the manufacturer's instructions. Enriched ECs were further FACS sorted with the surface marker profile $CD45^-LYVE1^-PDPN^-TER119^-CD31^+$.

Immune cell profiling

Single cell suspensions from lungs after erythrocyte lysis were stained with fluorescence-conjugated antibodies for CD45 (clone: 30-F11), CD11b (clone: M1/70) and Ly6G (clone: 1A8), together with blocking using the antibody against CD16/CD32 (Thermo Fisher Scientific, #14-0161-86).

EdU-based proliferation assay of lung endothelial cells

Pups at P7 were i.p. injected 150 µg of EdU in PBS, decapitated 3 hours later and lungs were dissected. Single cell suspensions were stained with fluorescence-conjugated antibodies [CD45 (clone: 30-F11) and CD31 (clone: MEC 13.3)]. Thereafter, EdU staining was performed using a Click-iT EdU assay kit (Thermo Fisher Scientific, #C10634) according to the manufacturer's instructions. $CD45^-CD31^+EdU^+$ cells were recognized as EdU^+ proliferative fraction of lung EC.

Adhesion of leukocytes

BEND3 cells overexpressing mTie1 constructs were seeded, changed medium two days later without FBS and stimulated with mouse TNFα (100 ng/ml, BioLegend, #575202) for 1 hour. After washing with PBS, U937-GFP cells in RPMI 1640 medium with 10% FBS were seeded on top of BEND3 cells and cocultured for 1 hour. Non-attached U937-GFP cells were removed by washing with PBS after coculture. Remaining cells were trypsinized and stained with fluorescence-conjugated antibody [CD31 (clone: MEC 13.3)]. $CD31^+GFP^-$ cells and $CD31^-GFP^+$ cells were recognized as BEND3 cells and U937-GFP cells, respectively.

Adhesion of tumor cells

BEND3 cells overexpressing mTie1 constructs were seeded. Two days later, LLC-RFP cells in DMEM medium with 10% FBS were seeded on top of BEND3 cells and cocultured for 1 hour. Non-attached LLC-RFP cells were removed by washing with PBS after coculture. Remaining cells were trypsinized and stained with fluorescence-conjugated antibody [CD31 (clone: MEC 13.3)]. $CD31^{+}RFP^{-}$ cells and $CD31^{-}RFP^{+}$ cells were recognized as BEND3 cells and LLC-RFP cells, respectively.

Transmigration of leukocytes

BEND3 cells overexpressing mTie1 constructs were seeded on top of Transwells with 3.0 µm pore (Corning, #3415) coated with 0.1% gelatin, changed medium two days later without FBS and stimulated with mouse TNFα (100 ng/ml, BioLegend, #575202) for 1 hour. After washing with PBS, U937-GFP cells in RPMI 1640 medium with 10% FBS were seeded on top of BEND3 cells. RPMI 1640 medium with 10% FBS were added in the lower chamber and cocultured for 6 hours. Transmigrated U937-GFP cells were collected from the lower chamber and stained with fluorescence-conjugated antibody [CD31 (clone: MEC 13.3)]. No transmigration of BEND3 cells was confirmed by no $CD31^{+}$ cells in the lower chamber.

After staining with the above-mentioned protocols (see antibody details in Table S3), doublets were excluded based on forward and side scatter, dead cells were excluded by FxCycle Violet staining (Thermo Fisher Scientific, #F10347) and cell numbers per mg tissue or per well were evaluated based on counting beads (Thermo Fisher Scientific, #C36950). Stained cells were analyzed and sorted using BD FACSCanto™ II, BD FACSAria™ III, BD FACSAria™ Fusion, and BD FACSMelody™. The distribution of each population and the frequency of individual cell populations were analyzed with FlowJo software (RRID:SCR_008520).

<u>**Immunofluorescence stainings**</u>

IF staining of cryo-sections

Tissues were embedded in cryomolds with OCT embedding matrix and were cut into 3 or 8 µm sections. Tissue sections were fixed in ice-cold methanol or 4% PFA for 10 minutes and were blocked using blocking buffer (5% FBS in PBS) for 45 minutes. The tissue sections were then incubated with primary antibodies against FOXO1 (Cell Signaling, #2880), CD31 (R and D systems, #AF3628) (BioLegend, #102502), Vcam1 (R and D systems, AF643), CD324/E-Cadherin (Cell Signaling, #3195), Ly6G (BioLegend, #127601), CD45-PE (BioLegend, #103106) and/or Ly6G-APC (BioLegend, #127614) in blocking buffer at r.t. for 1 hour or overnight at 4°C. Staining with fluorescence-conjugated corresponding secondary antibodies (Invitrogen, #A-32816, #A-78947, #A-31572 and #A-21206), together with Hoechst 33342 (Thermo Fisher Scientific, #H1399) was performed at r.t. for 45 minutes if necessary, and mounted with fluorescent mounting medium (Dako, #S302380-2). For FOXO1 and ERG staining, the tissue sections were stained with Alexa Fluor™ 647-conjugated ERG (abcam, #ab196149) together with Hoechst 33342 for 1 hour after staining with secondary antibody for FOXO1 and mounted with fluorescent mounting medium. Images were taken using Zeiss AxioScan slide scanner or Zeiss LSM 800 Confocal Laser Scanning Microscope, and image analysis was performed using ImageJ (RRID:SCR_003070).

IF staining of paraffin sections

Primary tumors were fixed with 4% PFA overnight at 4°C, embedded in paraffin and were cut into 5 µm sections. Tissue sections were deparaffinized with ROTICLEAR (Carl Roth, #A538.5) and serial ethanol solutions. The tissue sections were subjected to antigen retrieval with Target Retrieval

Solution (Agilent, #S169984-2) and were blocked using blocking buffer (5% FBS in PBS) at r.t. for 45 minutes. Afterwards, the tissue sections were then incubated at r.t. for 1 hour with primary antibodies against CD31 (R and D systems, #AF3628) and Desmin (abcam, #ab15200) in blocking buffer. Staining with fluorescence-conjugated corresponding secondary antibodies (Invitrogen, #A-21447 and #A-31572), together with Hoechst 33342 (Thermo Fisher Scientific, #H1399) was performed at r.t. for 1 hour, and mounted with fluorescent mounting medium (Dako, #S302380-2). Images were taken using Zeiss AxioScan slide scanner, and image analysis was performed using ImageJ (RRID:SCR_003070). Vessel density and vessel area are quantified as the particle number and area of $CD31^+$ signals in a region of interest (ROI), respectively. Desmin coverage was calculated based on the overlap area of $Desmin^+$ signals over $CD31^+$ signals. The Hematoxylin & Eosin (H&E) stainings were done on paraffin-embedded tissue slides by the General Core Equipment unit of the Medical Faculty Mannheim of Heidelberg University according to their standard operating procedures. All stained slides were scanned using an Axioscan 7 system.

Whole mount IF staining of mouse ear skin

Mouse ears were harvested and split into dorsal and ventral halves. The ear cartilage was carefully removed, and the dorsal ear skin was retained for further steps. Ear skin samples were fixed in 4% PFA for 30 minutes at r.t., followed by three times wash in PBS.Then the ear skin samples were blocked in blocking buffer (PBS containing 0.5% Triton X-100, 1% FBS, 3% BSA and 0.01% sodium deoxycholate) for 30 minutes at r.t.. Samples were then incubated with anti-mouse Lyve-1 (R and D systems, #MAB2125) diluted in blocking buffer overnight at 4°C. The following day, samples were washed three times in PBS for and incubated with fluorophore-conjugated secondary antibodies (Invitrogen, #A78946) for 4 hours at r.t. in the dark. After secondary antibody incubation, samples were washed three times with PBS and mounted for imaging. Images were acquired using Zeiss LSM 800 confocal microscope, then quantified by ImageJ.

Transmigration of tumor cells

BEND3 cells overexpressing mTie1 constructs were seeded on top of Transwells with 8.0 µm pore (Corning, #3422) coated with 0.1% gelatin. Two days later, LLC cells were resuspended in PBS, labeled with CellTrace CFSE Cell Proliferation Kit (Invitrogen, #C34554) according to the manufacturer's instructions and prepared in DMEM medium without FBS. After washing BEND3 cells with PBS, CFSE-labeled LLC cells were seeded on top of BEND3 cells. DMEM medium with 10% FBS were added in the lower chamber to make FBS gradient and cocultured for 6 hours. Both the upper and lower sides of the Transwells were washed with PBS and fixed with 4% PFA for 20 minutes at r.t. Transmigrated LLC cells were imaged on the lower side of the Transwells using THUNDER Imager DMi8 and image analysis was performed using ImageJ (RRID:SCR_003070). All the transmigrated LLC cells were found exclusively on the lower side of the Transwells.

Bulk RNA-sequencing of lung endothelial cells and data analysis

Lung ECs were isolated from Tie1-WT and Tie1-WT mice and total RNA was isolated using Arcturus PicoPure RNA isolation kit (Thermo Fisher Scientific, #KIT0204) according to the manufacturer's instructions. Quality control was performed by Bioanalyzer (Agilent) measurements. The sequencing library was generated with 50-100 ng of total RNA using the SMARTer Ultra Low RNA kit for Illumina sequencing (Clontech) according to the manufacturer's protocol. Sequencing reads were generated on the HiSeq4000 (50 bp Single-End; Illumina) with 4-8 samples per lane. The sequenced reads were aligned to the mouse reference genome mm10 using STAR aligner (v.2.7.11a) (4). Differential gene expression analysis was computed using DEseq2 (v.3.18) (5) with default settings and pre-filtering

parameters. Only transcripts with a TPM ≥ 1 in at least one sample were considered for the downstream analysis. EnrichR, Gene Set Enrichment Analysis (GSEA) (6) and Ingenuity Pathway Analysis (IPA, Qiagen, RRID:SCR_008653) were undertaken to study regulated molecular pathways and corresponding biological functions.

Gene expression analysis by quantitative PCR

Total RNA was isolated using Arcturus PicoPure RNA isolation kit and was transcribed into cDNA using the Quantitect reverse transcription kit (Qiagen, #205313). Quantitative PCRs were performed with Taqman master mix (Thermo Fisher Scientific, #4444965) and Taqman probes of target genes; *Actb* (Mm00607939_s1, Life Technologies, #4331182), *Icam1* (Mm00516023_m1, Life Technologies, #4331182), *Il6* (Mm01210733_m1, Life Technologies, #4331182) and *Vcam1* (Mm01320970_m1, Life Technologies, #4331182). Gene expression levels were calculated based on the ΔΔCt relative quantification method. mRNA levels were normalized to Actin Beta (*Actb)* expression.

Protein isolation

Snap-frozen tissue samples were dissociated with metal beads to prepare tissue powder and lysed with RIPA-based lysis buffer containing protease inhibitors (Protease-Inhibitor-Mix HP, SERVA, #39106.03) and PhosSTOP (Roche, #4906845001). Alternatively, tissue samples were homogenized and lysed with metal beads in RIPA-based lysis buffer containing protease inhibitors and 2 mM Sodium orthovanadate (Na_3VO_4, Carl Roth, #0735.2). For cultured cells, supernatant was collected and cells were lysed using RIPA-based lysis buffer containing protease inhibitors and 2 mM Na_3VO_4. Lysates were cleared by centrifugation and used for immunoprecipitation or boiled with 5x Laemmli buffer for IB.

Immunoprecipitation

Tissue lysates were precleared by incubation with protein G-sepharose beads (GE Healthcare, #17-0618-01) at 4°C on a rotator for 2 hours and subjected to immunoprecipitation. For immunoprecipitation, protein G-sepharose beads were incubated with 0.5-3 µg antibody at 4°C on a rotator for 1 hour. Lysates or plasma samples were incubated with antibody-incubated beads at 4°C on a rotator for 2 hours. Beads were washed with RIPA-based lysis buffer containing 2 mM Na_3VO_4 and boiled with 2x Laemmli buffer.

ELISA

TNF-α and IL-6 levels were quantified from the serum samples with/out LPS challenge using mouse ELISA kits (R and D systems, #MTA00B-1 and #M6000B-1, respectively). The samples with LPS challenge were diluted in order to fit within the range of the standard curve.

Immunoblot

Immunoprecipitates and/or lysates were separated by SDS-PAGE followed by Immunoblot. Blots were probed with the indicated antibodies in the figures, and were stripped with ReBlot Plus Strong Antibody Stripping Solution (Millipore, #2504) when necessary. The signal intensity of each band was quantified using ImageJ (RRID:SCR_003070).

Statistics

Preparation of the graphs and corresponding statistical analysis were performed using GraphPad Prism version 9.4.1 (RRID:SCR_002798). Data are expressed as the mean ± SD in dot plots. Employed statistical tests are Mann-Whitney U-test (comparison between Tie1-WT and Tie1-CR only), two-way ANOVA followed by Šídák's multiple comparison test (comparison between Tie1-WT and Tie1-CR with

challenge/treatment) or Log-rank (Mantel-Cox) test (survival). A p-value of less than 0.05 was considered as statistically significant. ****$p < 0.0001$; ***$p < 0.001$; **$p < 0.01$; *$p < 0.05$.

Data availability

Bulk RNAseq data of lung ECs from Tie1-WT and Tie1-CR mice have been deposited to GEO under accession number GSE287671 (Reviewer's token – ifwhygoujvwvfaz). Uncropped Western blot images are archived as a separate supplemental file.

References:

1. Yabkowitz R, Meyer S, Black T, Elliott G, Merewether LA, and Yamane HK. Inflammatory cytokines and vascular endothelial growth factor stimulate the release of soluble tie receptor from human endothelial cells via metalloprotease activation. *Blood.* 1999;93(6):1969-79.
2. Singhal M, Gengenbacher N, Abdul Pari AA, Kamiyama M, Hai L, Kuhn BJ, et al. Temporal multi-omics identifies LRG1 as a vascular niche instructor of metastasis. *Sci Transl Med.* 2021;13(609):eabe6805.
3. Karkkainen MJ, Saaristo A, Jussila L, Karila KA, Lawrence EC, Pajusola K, et al. A model for gene therapy of human hereditary lymphedema. *Proc Natl Acad Sci U S A.* 2001;98(22):12677-82.
4. Dobin A, Davis CA, Schlesinger F, Drenkow J, Zaleski C, Jha S, et al. STAR: ultrafast universal RNA-seq aligner. *Bioinformatics.* 2013;29(1):15-21.
5. Love MI, Huber W, and Anders S. Moderated estimation of fold change and dispersion for RNA-seq data with DESeq2. *Genome Biol.* 2014;15(12):550.
6. Subramanian A, Tamayo P, Mootha VK, Mukherjee S, Ebert BL, Gillette MA, et al. Gene set enrichment analysis: a knowledge-based approach for interpreting genome-wide expression profiles. *Proc Natl Acad Sci U S A.* 2005;102(43):15545-50.

Supplemental figures and tables

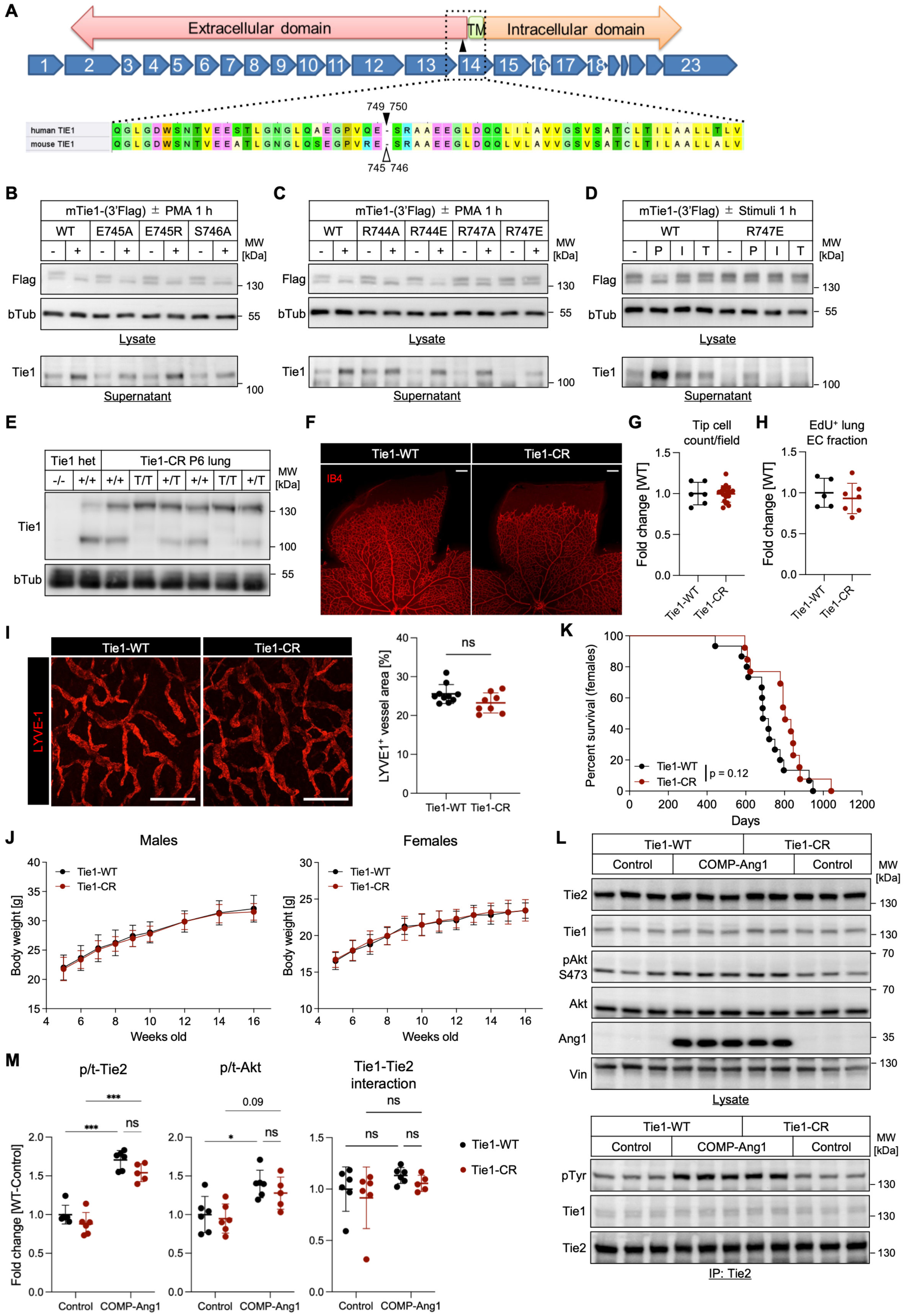

Supplemental Figure 1. Tie1 cleavage is dispensable for vascular growth and Tie1-CR mice display no overt physiological defects. (**A**) Schematic depiction of different domains of the Tie1 protein. Blue boxes show exons with their corresponding number. Amino acid sequence homology between human Tie1 and mouse Tie1 of the marked peptide sequence is shown. The cleavage site is indicated with arrowheads. TM: trans-membrane domain. (**B**-**D**) IB analysis of mTie1 cleavage. Cells overexpressing Flag-tagged mTie1 (either WT or mentioned point mutants) were treated with PMA (P; 10 ng/ml), IL-1β (I; 100 ng/ml), or TNFα (T; 100 ng/ml) for 1 hour. Ectopically expressed full-length mTie1 was detected in the lysates using an anti-Flag antibody. Tie1-ECD was detected in the cell supernatant using an anti-Tie1 antibody raised against Tie1-ECD after immunoprecipitation with the same antibody. bTub: beta-Tubulin. (**E**) IB of lung lysates from homozygous (T/T) and heterozygous (+/T) targeted mutation-bearing Tie1-CR mice, and WT (+/+) mice at P6 born from heterozygous breeders. Lung lysates from constitutive Tie1 KO (-/-) and WT (+/+) embryos (born from heterozygous Tie1-deleted [+/-] breeders) were included as controls. (**F**) Representative whole-mount images of retinas stained with EC marker IB4 at P6 of the pups born from heterozygous breeders. Scale bar: 200 µm. (**G**) Quantification of tip cell ($IB4^+ESM1^+$) numbers in retinas. Tie1-WT mice: *n* = 6; Tie1-CR mice: *n* = 18. (**H**) EdU^+ proliferative fraction of lung ECs at P7 of the pups born from heterozygous breeders. Tie1-WT mice: *n* = 5; Tie1-CR mice: *n* = 7. (**I**) Representative images (left) and quantitation (right) of LYVE1+ vessel area in ear skin tissues of Tie1-CR and Tie1-WT mice. Tie1-WT mice: *n* = 10; Tie1-CR mice: *n* = 8. Scale bar: 20 µm. (**J**) Body weight records of male and female mice over time. For males, Tie1-WT mice: *n* = 10; male Tie1-CR mice: *n* = 8. For Females, *n* = 6 mice per genotype. (**K**) Kaplan-Meier plot displaying overall survival in the female aging cohort. Tie1-WT mice: *n* = 15; Tie1-CR mice: *n* = 13. (**L and M**) Tie1-CR and Tie1-WT mice were injected with COMP-Ang1 or control AAV injection. (**L**) IB of whole lung lysates (top) and Tie2-immunoprecipitates (Tie2-IP, bottom). In Tie2-IP samples, subsequent IB with pTyr antibody marks phosphorylated Tie2 and Tie1 IB reflects Tie2-interacting Tie1. Ang1: COMP-Ang1; Vin: Vinculin. (**M**) Quantification of Tie2 and Akt phosphorylation and Tie1-Tie2 interaction in corresponding immunoblots from either Tie2-IP or lung lysates following COMP-Ang1 or control AAV injection. *n* = 5-6. Data are represented as mean ± SD (**G, H, I, J, and M**). ****p* < 0.001; **p* < 0.05. Mann-Whitney U-test (**G-I**), two-way ANOVA followed by Šídák's multiple comparison test (**M**) and Log-rank (Mantel-Cox) test (**K**).

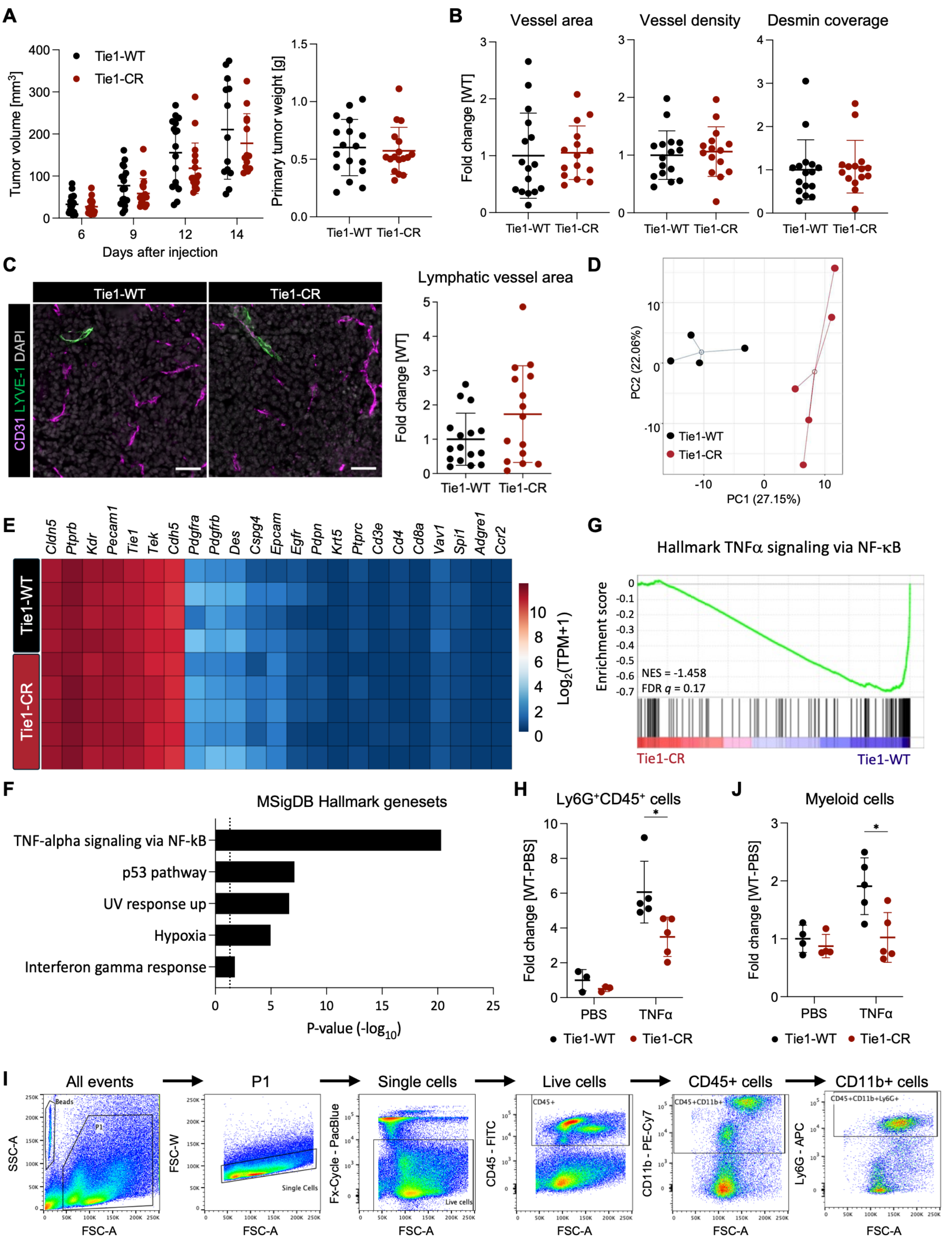

Supplemental Figure 2. Tie1 cleavage does not affect tumor angiogenesis but evokes vascular response upon inflammatory stimulation. (**A**) The kinetics of primary tumor growth (left) and the final tumor weight (right) in s.c. LLC-bearing mice. *n* = 17 each. (**B**) Quantification of vessel area, vessel density, and pericyte coverage of LLC tumor vasculature. Tie1-WT mice: *n* = 16; Tie1-CR mice: *n* = 15. (**C**) Representative IF images (left) and quantification (right) of lymphatic vessel area across LLC tumors grown in Tie1-CR and Tie1-WT mice. Tie1-WT mice: *n* = 16; Tie1-CR mice: *n* = 15. Scale

bar: 50 µm. (**D**) PCA plot of Tie1-WT and Tie1-CR lung EC samples isolated for gene expression analysis. Tie1-WT mice: $n = 4$; Tie1-CR mice: $n = 5$. (**E**) Heatmap comparing transcriptomic reads of cell type-specific genes between Tie1-WT and Tie1-CR lung EC samples to verify the purity of sorted samples. The absolute values of log-transformed transcripts per million reads (TPM) are shown. (**F**) Bar plot showing enriched MSigDB Hallmark genesets in DEGs in Tie1-CR ECs as analyzed with EnrichR. The dashed line marks a P-value of 0.05. (**G**) Gene set enrichment plot showing a negative enrichment of the hallmark geneset "TNFa signaling via NF-κB" in Tie1-CR EC samples compared with Tie1-WT ECs. NES: normalized enrichment score; FDR: false discovery rate. (**H**) The quantification of Ly6G$^+$ myeloid cells in IF images (corresponding to Figure 1J). PBS: $n = 3$ each; TNFα: $n = 5$ each. (**I**) Stepwise gating strategy for FACS analysis of CD45$^+$CD11b$^+$Ly6G$^+$ myeloid cells in lungs. (**J**) FACS-based analysis of CD45$^+$CD11b$^+$ myeloid cells in lungs challenged with TNFα or control PBS for 6 hours. PBS: $n = 4$ each; TNFα: $n = 5$ each. Data are represented as mean ± SD (**A, B, C, H** and **J**). *$p < 0.05$. Mann-Whitney U-test (**A, B,** and **C**) and two-way ANOVA followed by Šídák's multiple comparison test (**H** and **J**).

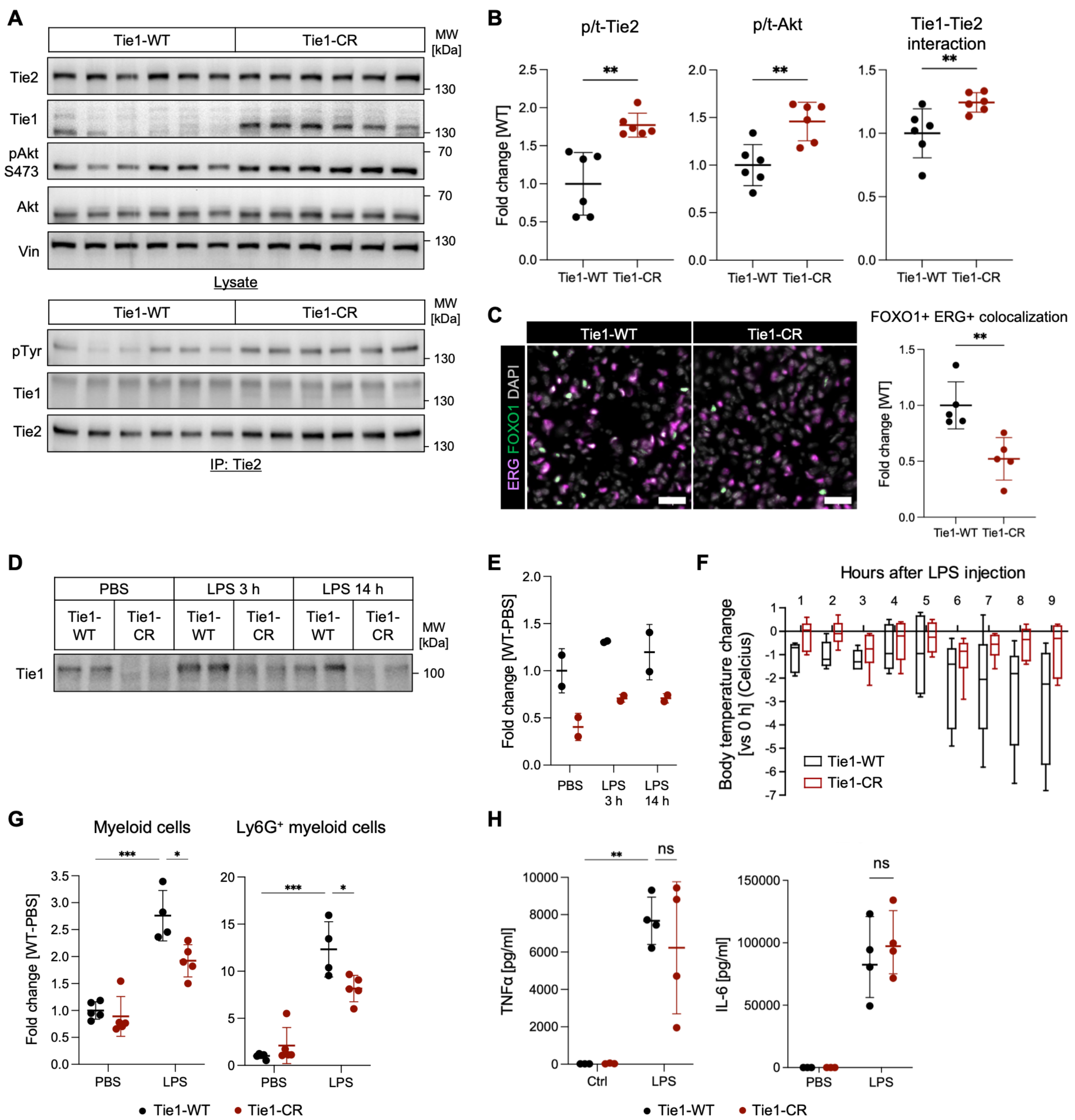

Supplemental Figure 3. Tie1-CR mice preserve Tie2 signaling after inflammatory challenge. (**A-C**) Lung tissues from TNFα-injected Tie1-CR and Tie1-WT mice were collected for analyzing Tie2 signaling. (**A**) IB of whole lung lysates (top) and Tie2-immunoprecipitates (Tie2-IP, bottom). In Tie2-IP samples, subsequent IB with pTyr antibody marks phosphorylated Tie2, and Tie1 IB reflects Tie2-interacting Tie1. Vin: Vinculin. (**B**) Quantification of Tie2 and Akt phosphorylation and Tie1-Tie2 interaction in corresponding immunoblots from either Tie2-IP or lung lysates following TNFα injections in Tie1-CR and Tie1-WT mice. n = 6 mice per genotype. (**C**) Representative images (left) and quantitation (right) of nuclear FOXO1 in lung ECs of TNFα-injected Tie1-CR and Tie1-WT mice. n = 5 mice per genotype. Scale bar: 20 µm. (**D** and **E**) IB (**D**) and corresponding quantification (**E**) of soluble Tie1-ECD in plasma from Tie1-WT or Tie1-CR mice challenged with LPS or PBS for 3 or 14 hours. n = 2 each. (**F**) Box plot showing the change in body temperature after i.p. injection of LPS. n = 6 each. (**G**) FACS-based analysis of myeloid cells in lungs challenged with LPS or PBS for 3 hours. PBS: n = 5 each; LPS Tie1-WT: n = 4; LPS Tie1-CR: n = 5. (**H**) Plasma levels of TNFα and IL-6 in Tie1-CR

and Tie1-WT mice challenged with LPS or PBS. PBS: $n = 3$ each; LPS: $n = 4$ each. Data are represented as mean ± SD (**B**, **C**, **E, G** and **H**). ***$p < 0.001$; **$p < 0.01$; *$p < 0.05$. Mann-Whitney U-test (**B** and **C**) and two-way ANOVA followed by Šídák's multiple comparison test (**G** and **H**).

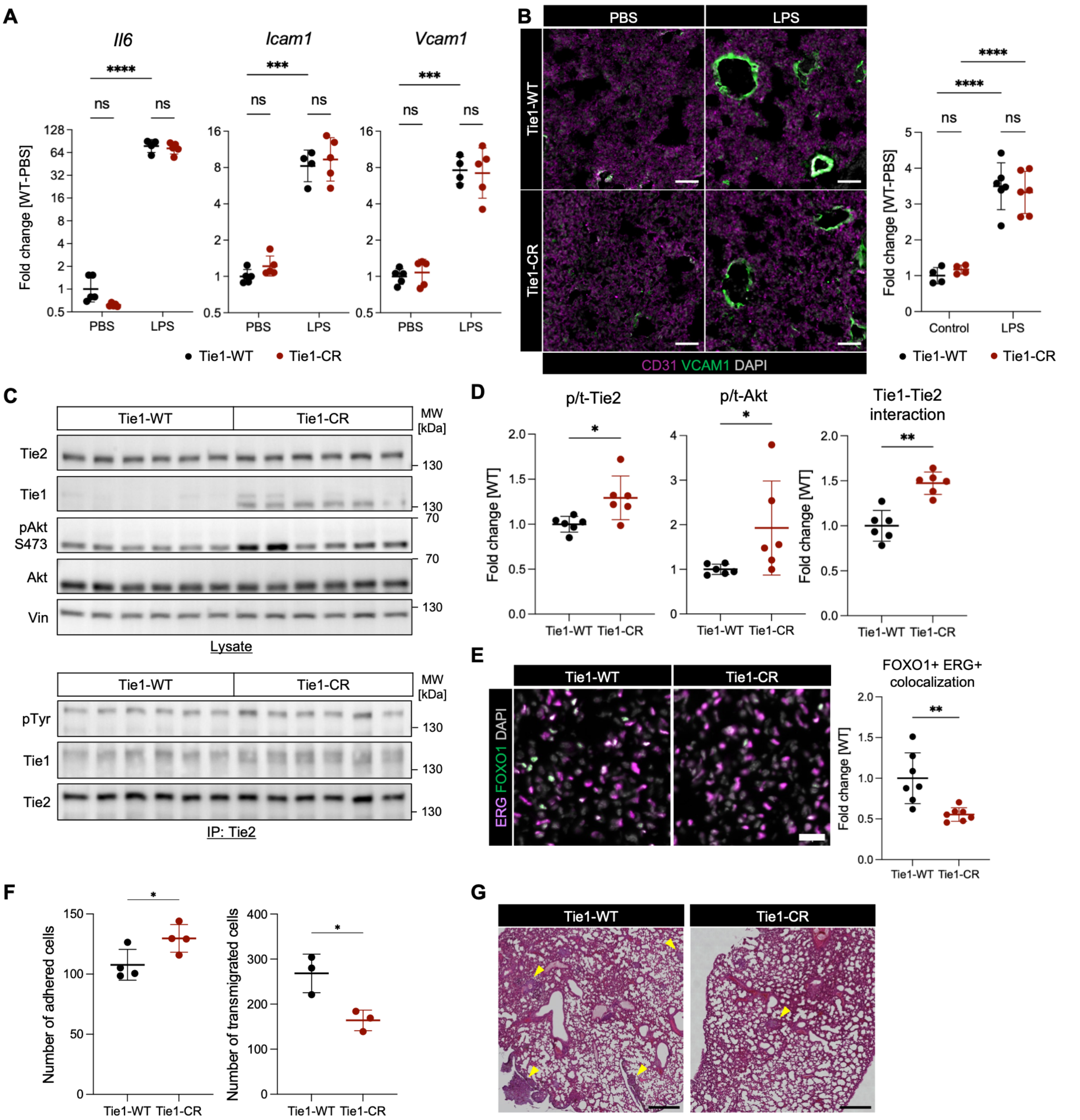

Supplemental Figure 4. Tie1-CR mice resist LPS-induced endotoxemia and transendothelial migration of tumor cells. (**A**) Expression analysis of selected genes in lung ECs from Tie1-WT or Tie1-CR mice challenged with LPS or PBS for 3 hours. PBS: *n* = 5 each; LPS Tie1-WT: *n* = 4; LPS Tie1-CR: *n* = 5. (**B**) Representative images (left) and quantitation (right) of VCAM1+ vessel area in lung tissues of Tie1-CR and Tie1-WT mice challenged with LPS or PBS for 3 hours. PBS: *n* = 4 each; LPS: *n* = 6 each. Scale bar: 50 µm. (**C-E**) Lung tissues from LPS-injected Tie1-CR and Tie1-WT mice were collected for analyzing Tie2 signaling. (**C**) IB of whole lung lysates (top) and Tie2-immunoprecipitates (Tie2-IP, bottom). In Tie2-IP samples, subsequent IB with pTyr antibody marks phosphorylated Tie2, and Tie1 IB reflects Tie2-interacting Tie1. Vin: Vinculin. (**D**) Quantification of Tie2 and Akt phosphorylation and Tie1-Tie2 interaction in corresponding immunoblots from either Tie2-IP or lung lysates following LPS injections in Tie1-CR and Tie1-WT mice. *n* = 6 mice per genotype. (**E**) Representative images (left) and quantitation (right) of nuclear FOXO1 in lung ECs of LPS-injected Tie1-CR and Tie1-WT mice. *n* = 7 mice per genotype. Scale bar: 20 µm. (**F**) Adhesion (left) and

transendothelial migration (right) of LLC cells. *n* = 4 (adhesion) and *n* = 3 (migration) replicates. (**G**) Representative H&E images of metastasis-bearing lung tissues of Tie1-CR and Tie1-WT mice (corresponding to Figure 1N). Yellow arrowheads mark tumor lesions. Scale bar: 50 µm. Data are represented as mean ± SD (**A**, **B, D**, **E,** and **F**). $****p < 0.0001$; $***p < 0.001$; $**p < 0.01$; $*p < 0.05$. Mann-Whitney U-test (**D, E,** and **F**) and two-way ANOVA followed by Šídák's multiple comparison test (**A** and **B**).

Table S1. Observations with humane endpoint criteria in aging cohort.

Gender	Genotype	Survival [days]	Observations
Male	Tie1-WT	370	bad state of health in general, euthanized
Male	Tie1-WT	683	open skin wound, scratches, euthanized
Male	Tie1-WT	685	found dead
Male	Tie1-WT	707	curved back, shrunk, euthanized
Male	Tie1-WT	743	stereotype, pronounced barbering, euthanized
Male	Tie1-WT	743	stereotype, pronounced barbering, euthanized
Male	Tie1-WT	748	found dead
Male	Tie1-WT	750	bad state of health in general, curved back, shrunk, crouched, euthanized
Male	Tie1-WT	766	sternum is clearly visible, euthanized
Male	Tie1-WT	778	curved back, shrunk, crouched, shaky gait, euthanized
Male	Tie1-WT	779	open skin wound, scratches, euthanized
Male	Tie1-WT	808	open skin wound, scratches, animal scratch its ears, euthanized
Male	Tie1-WT	849	curved back, shrunk, crouched, bad state of health in general, euthanized
Male	Tie1-WT	1059	curved back, shrunk, crouched, tumor, euthanized
Male	Tie1-CR	617	losing weight, euthanized
Male	Tie1-CR	625	found dead
Male	Tie1-CR	691	moribund, euthanized
Male	Tie1-CR	699	curved back, shrunk, crouched, bad state of health in general, euthanized
Male	Tie1-CR	707	open skin wound, scratches including neck, euthanized
Male	Tie1-CR	711	inflammatory eye problem, euthanized
Male	Tie1-CR	783	bad state of health in general, euthanized
Male	Tie1-CR	785	open skin wound, scratches, curved back, shrunk, crouched, euthanized
Male	Tie1-CR	787	bite wound, curved back, shrunk, crouched, euthanized
Male	Tie1-CR	847	curved back, shrunk, crouched, euthanized
Male	Tie1-CR	934	neurological deficit, abdomen increased in size, curved back, shrunk, crouched, euthanized
Male	Tie1-CR	952	bad state of health in general, curved back, shrunk, crouched, euthanized
Female	Tie1-WT	442	found dead
Female	Tie1-WT	578	found dead
Female	Tie1-WT	605	eye problem, bad state of health in general, euthanized
Female	Tie1-WT	613	curved back, shrunk, crouched, euthanized
Female	Tie1-WT	682	slight head tilt, slight ataxia, euthanized
Female	Tie1-WT	684	found dead
Female	Tie1-WT	687	open skin wound, scratches, euthanized
Female	Tie1-WT	688	tumor on the upper arm, euthanized
Female	Tie1-WT	717	found dead

Female	Tie1-WT	721	open skin wound, scratches, large, deep scratch wounds on its neck and behind its ear, euthanized
Female	Tie1-WT	750	open skin wound, scratches, euthanized
Female	Tie1-WT	777	found dead
Female	Tie1-WT	796	breathing problems, mild ataxia, weight loss, euthanized
Female	Tie1-WT	927	tumor on the leg, euthanized
Female	Tie1-WT	947	curved back, shrunk, crouched, euthanized
Female	Tie1-CR	595	swollen belly, curved back, shrunk, crouched, euthanized
Female	Tie1-CR	608	curved back, shrunk, crouched, open skin wound, scratches, euthanized
Female	Tie1-CR	623	bad state of health in general, euthanized
Female	Tie1-CR	778	bad state of health in general, tumor, euthanized
Female	Tie1-CR	792	eye problems, weight loss, bad state of health in general, curved back, shrunk, crouched, euthanized
Female	Tie1-CR	792	eye problems, weight loss, bad state of health in general, curved back, shrunk, crouched, euthanized
Female	Tie1-CR	803	thick belly with tumor, euthanized
Female	Tie1-CR	833	tumor, increase in circumference on the upper lip, euthanized
Female	Tie1-CR	844	increased circumference of the neck, euthanized
Female	Tie1-CR	845	neurological deficit, has a big belly and drags one hind leg, tumor, curved back, shrunk, crouched, euthanized
Female	Tie1-CR	877	curved back, shrunk, crouched, euthanized
Female	Tie1-CR	880	neurological deficit, ataxia, euthanized
Female	Tie1-CR	1041	curved back, shrunk, crouched, euthanized

Table S2. List of bacterial and virus strains used in this study.

Strain	Source	Cat#
pLV-mTie1-WT-3'Flag	Vector Builder	Cat#VB221020-1212bym
pLV-mTie1-E745A-3'Flag	Vector Builder	Cat#VB230225-1043vjq
pLV-mTie1-E745R-3'Flag	Vector Builder	Cat#VB230619-1353vqb
pLV-mTie1-S746A-3'Flag	Vector Builder	Cat#VB230225-1045kmx
pLV-mTie1-R744A-3'Flag	Vector Builder	Cat#VB230225-1041kdc
pLV-mTie1-R744E-3'Flag	Vector Builder	Cat#VB221105-1070ecy
pLV-mTie1-R747A-3'Flag	Vector Builder	Cat#VB230225-1046mnu
pLV-mTie1-R747E-3'Flag	Vector Builder	Cat#VB230510-1417asp

Table S3. List of antibodies used in this study

Antibody	Source	Cat#, RRID
Phospho-Akt Ser473 (D9E)	Cell Signaling	Cat#4060; RRID: AB_2315049
Akt (pan) (40D4)	Cell Signaling	Cat#2920; RRID: AB_1147620
Ang1	R and D systems	Cat#AF923
bTubulin	abcam	Cat#ab6046; RRID: AB_2210370
CD11b-PE-Cy7 (clone: M1/70)	BioLegend	Cat#101216; RRID: AB_312799
CD31	R and D systems	Cat#AF3628; RRID: AB_2161028
CD31 (clone: MEC 13.3)	BioLegend	Cat#102502; RRID:AB_312909
CD31-APC (clone: MEC 13.3)	BioLegend	Cat#102510; RRID: AB_312917
CD31-FITC (clone: 390)	BD Pharmingen	Cat#558738; RRID: AB_397097
E-Cadherin (CD324, 24E10)	Cell Signaling	Cat#3195; RRID: AB_2291471
CD45-FITC (clone: 30-F11)	BioLegend	Cat#103108; RRID: AB_312973
CD45-PE (clone: 30-F11)	BioLegend	Cat#103106; RRID: AB_312971
Desmin	abcam	Cat#ab15200; RRID: AB_301744
ERG, Alexa Fluor™ 647	abcam	Cat# ab196149; RRID: AB_3697463
ESM1	R and D systems	Cat#AF1999; RRID: AB_2101810
Flag (clone: M2)	Sigma-Aldrich	Cat#F1804; RRID: AB_262044
FOXO1	Cell Signaling	Cat#2880; RRID:AB_2106495
LYVE1	R and D systems	Cat#MAB2125; RRID: AB_2138528
LYVE1-PE (clone: ALY7)	eBioscience	Cat#12-0443-82; RRID: AB_2802179
Ly6G (clone: 1A8)	BioLegend	Cat#127601; RRID: AB_1089180
Ly6G-APC (clone: 1A8)	BioLegend	Cat#127614; RRID: AB_1877163
NG2	Millipore	Cat#AB5320; RRID: AB_11213678
PDPN-PE (clone: eBio8.1.1)	eBioscience	Cat#12-5381; RRID: AB_1907440
TER119-PE (clone: TER-119)	BD Pharmingen	Cat#553673; RRID: AB_394986
Tie1	R and D systems	Cat#AF619; RRID: AB_355481
Tie2	R and D systems	Cat#AF762; RRID: AB_2203220
Phospho-tyrosine (clone: 4G10)	Merck	Cat#05-321; RRID: AB_309678
Vcam1 (CD106)	R and D systems	Cat#AF643; RRID: AB_355499
Vinculin antibody (EPR8185)	abcam	Cat#ab129002; RRID: AB_11144129
Rabbit anti-Goat Ig-HRP	Dako	Cat#P0160
Rabbit anti-Goat IgG-HRP	Invitrogen	Cat#31402; RRID: AB_228395
Goat anti-Mouse IgG-HRP	BioRad	Cat#1721011; RRID: AB_2617113
Goat anti-Rabbit Ig-HRP	Dako	Cat#P0448; RRID: AB_2617138
Goat anti-Rabbit IgG-HRP	Invitrogen	Cat#31460; RRID: AB_228341
Donkey anti-Goat IgG, Alexa Fluor™ Plus 555	Invitrogen	Cat#A-32816; RRID: AB_2762839
Donkey anti-Goat IgG, Alexa Fluor™ 647	Invitrogen	Cat#A-21447; RRID: AB_141844
Donkey anti-Rabbit IgG, Alexa Fluor™ 488	Invitrogen	Cat#A-21206; RRID: AB_2535792
Donkey anti-Rabbit IgG, Alexa Fluor™ 555	Invitrogen	Cat#A-31572; RRID: AB_162543
Donkey anti-Rat IgG, Alexa Fluor™ 568	Invitrogen	Cat#A-78946; RRID: AB_2910653
Donkey anti-Rat IgG, Alexa Fluor™ 647	Invitrogen	Cat#A-78947; RRID: AB_2910635
