# Supplementary material for: Tie1 ectodomain cleavage governs vascular responses to inflammatory stimulation but is dispensable for angiogenic signaling
Source: J Clin Invest. 2026 Oct 1;136(19):e204085. doi: 10.1172/JCI204085 (PMC13626837; doi:10.1172/JCI204085)

Full unedited blots for Main Figure 1

H

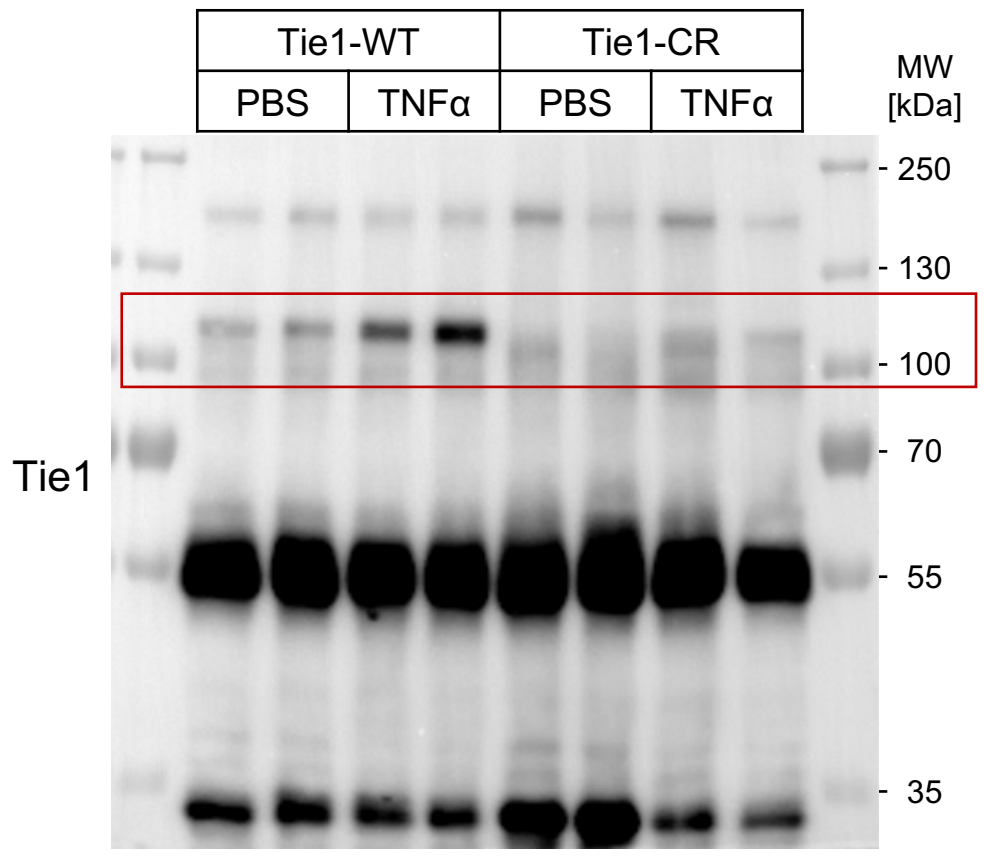

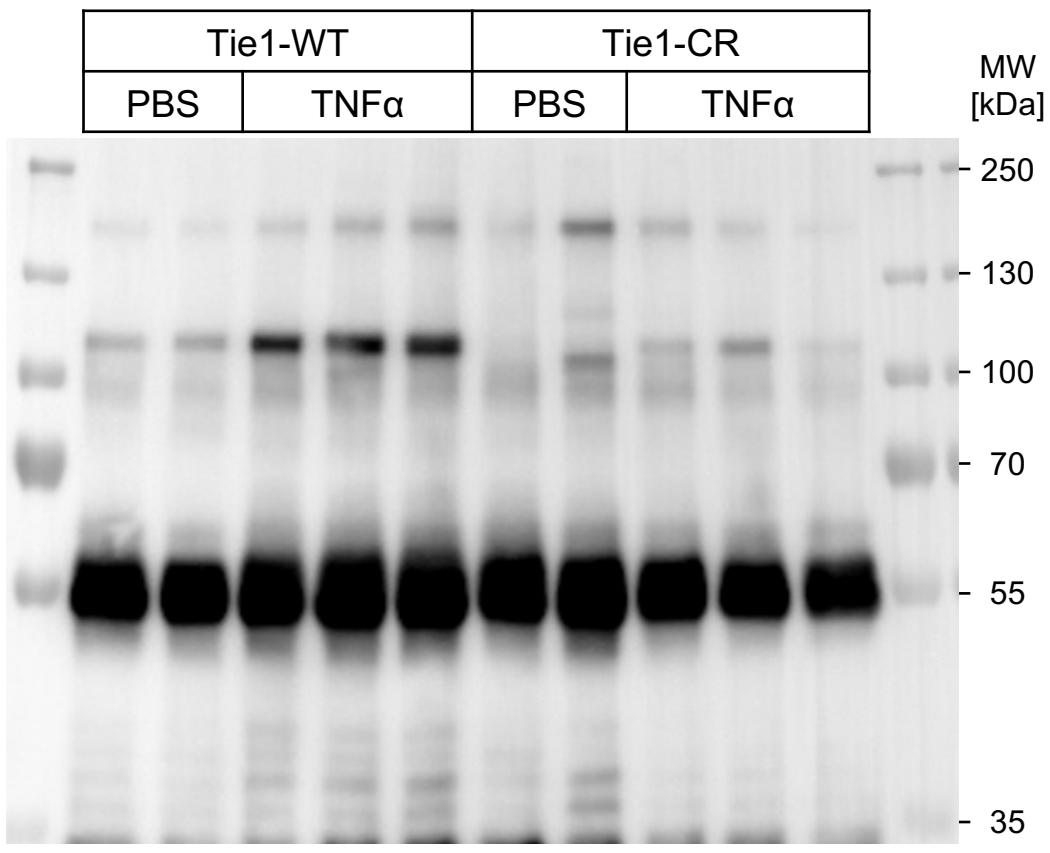

Full unedited blots for Supplemental Figure 1

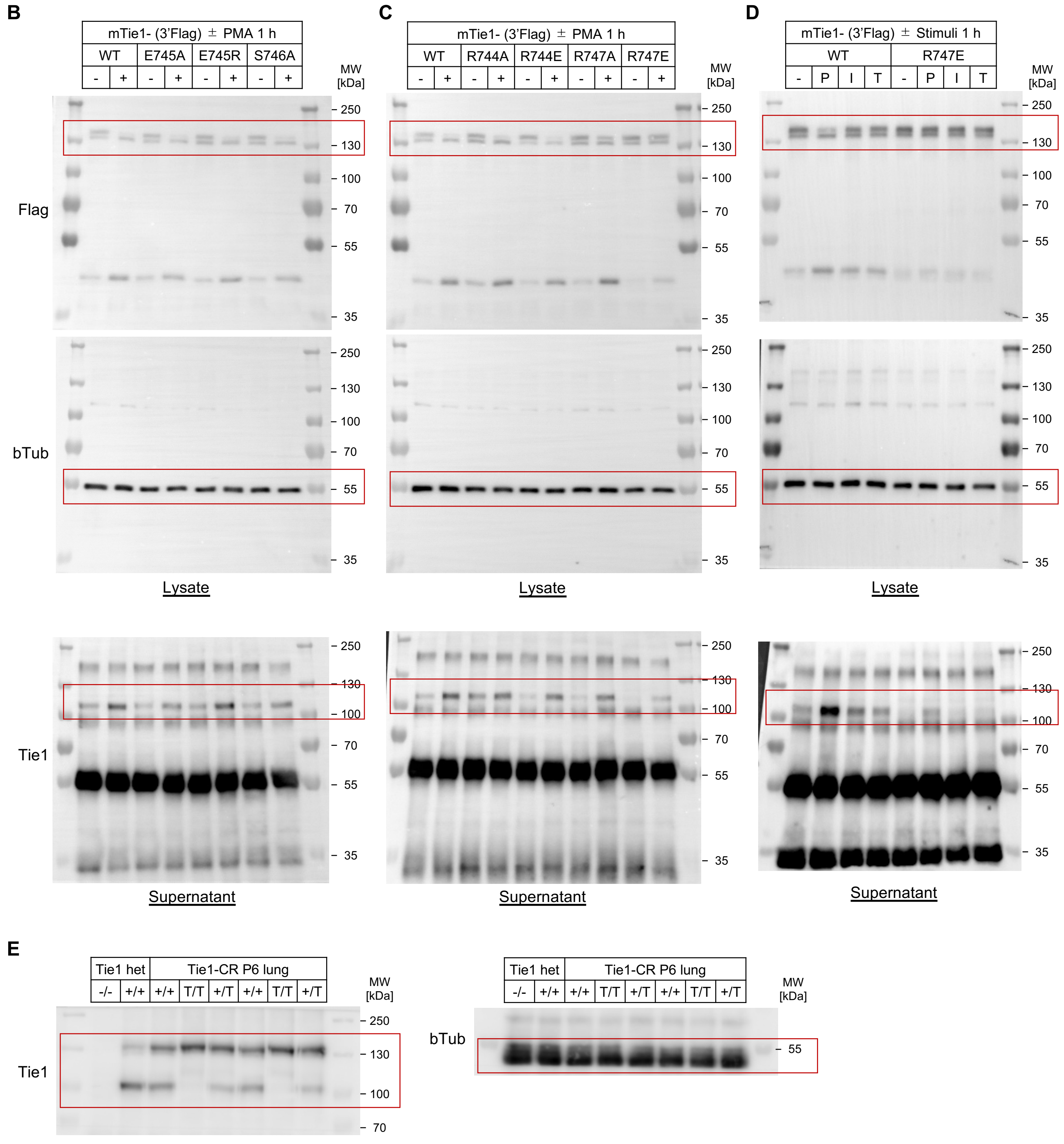

Full unedited blots for Supplemental Figure 1 continued

L

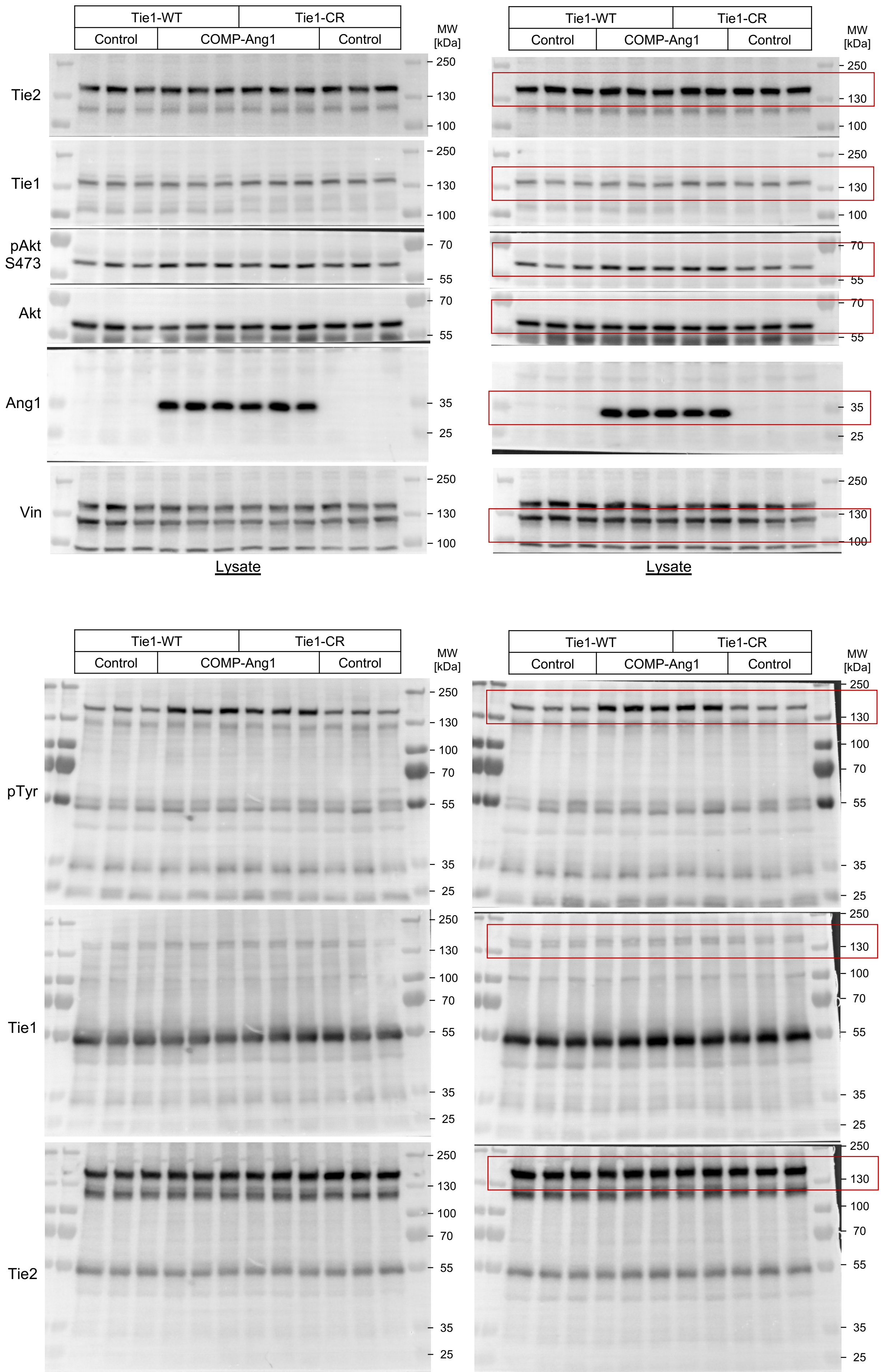

Full unedited blots for Supplemental Figure 3

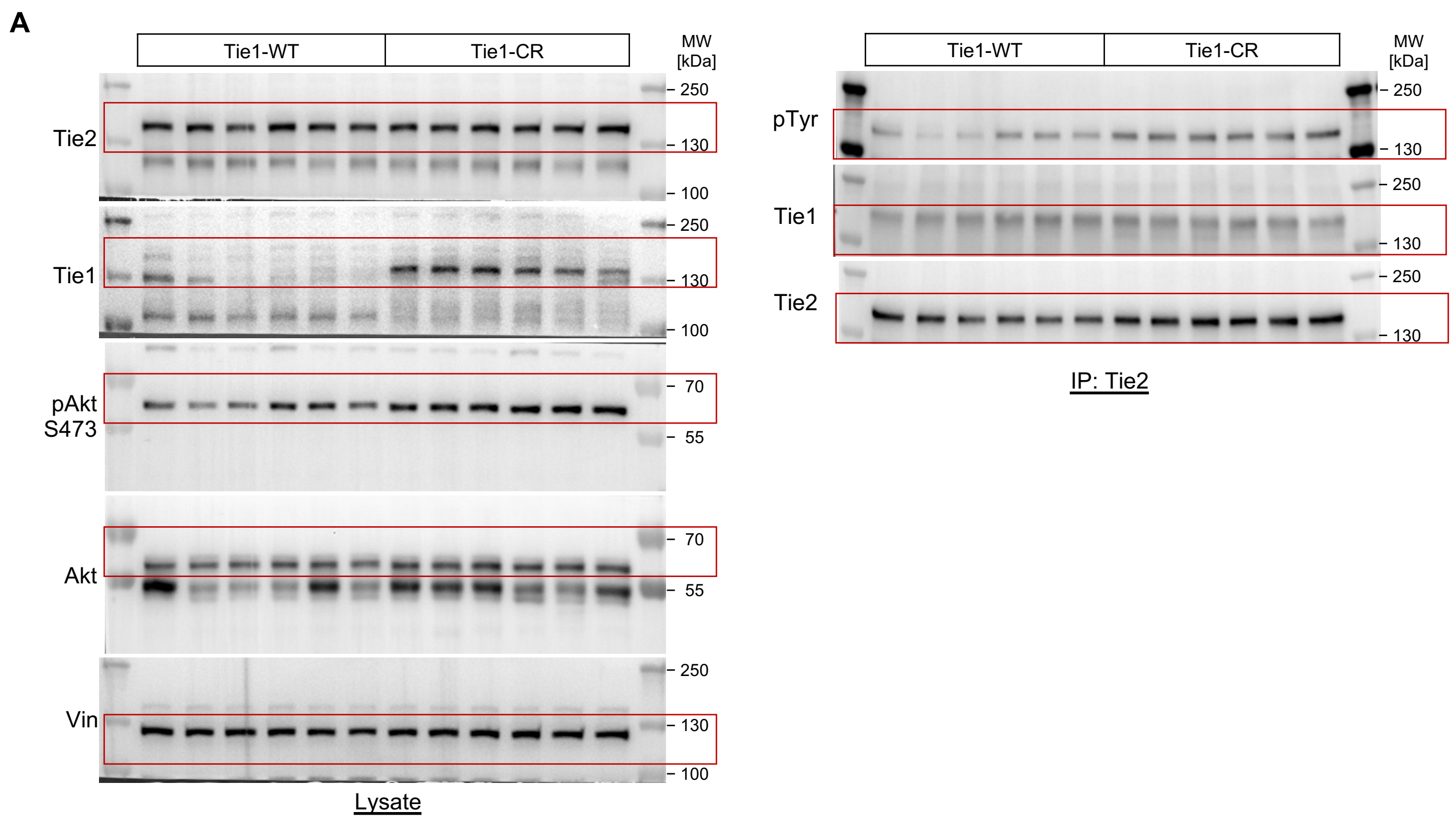

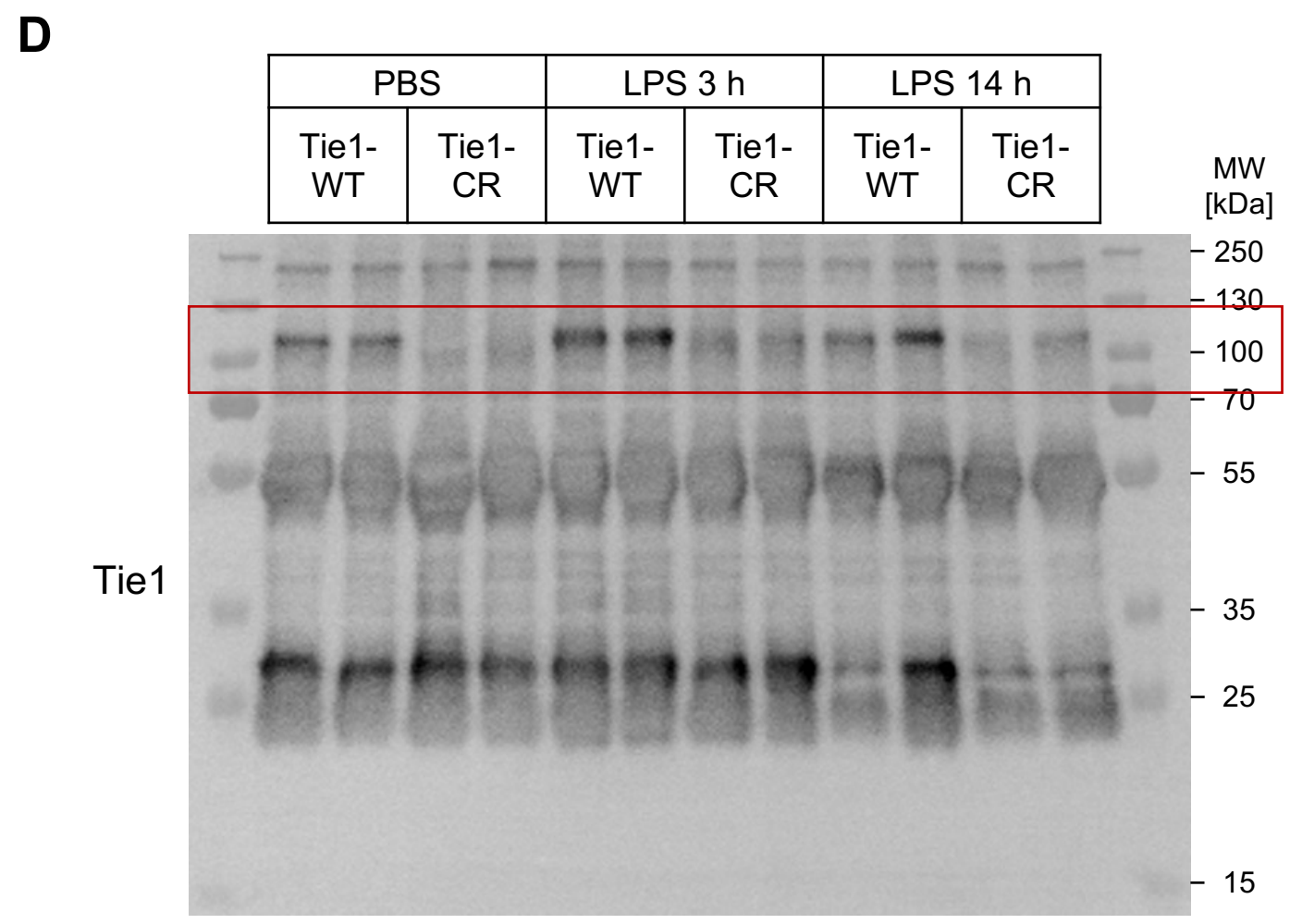

Full unedited blots for Supplemental Figure 4

C

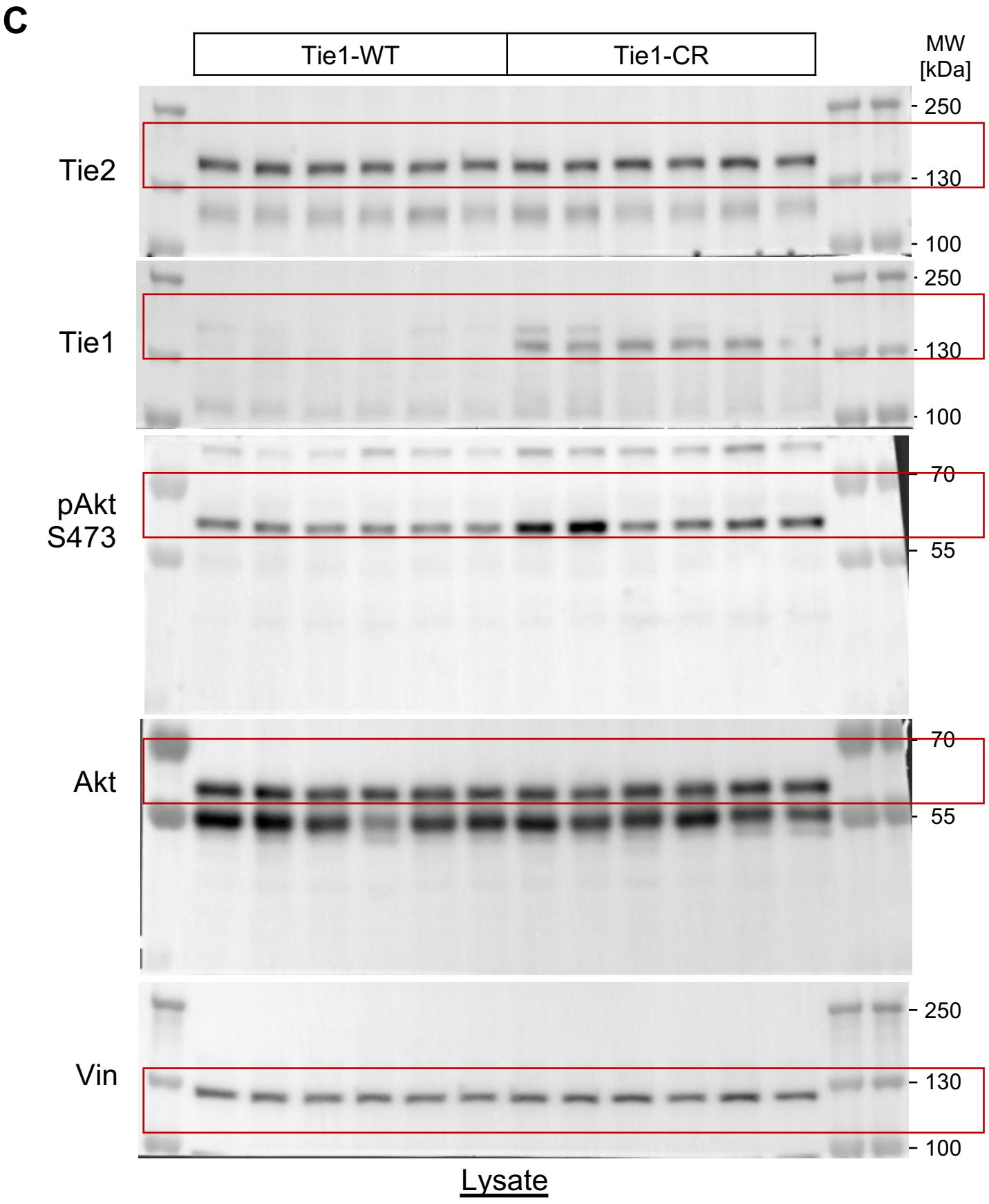

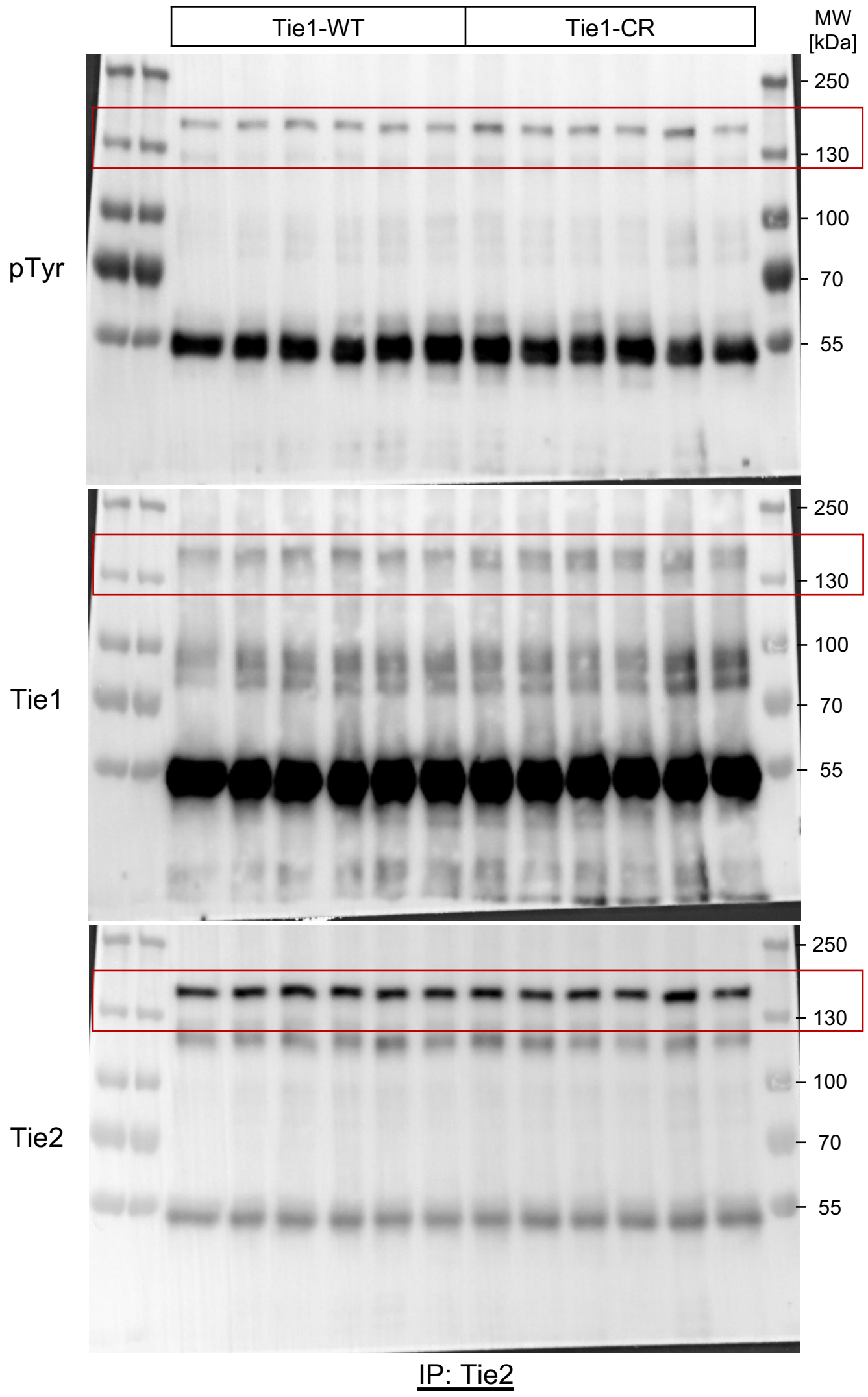

Supplement: Supplementary file 2 [file jci-136-204085-s354.pdf]
